# A Semantic Relatedness Model for the Automatic Cluster Analysis of Phonematic and Semantic Verbal Fluency Tasks Performed by People With Parkinson Disease: Prospective Multicenter Study

**DOI:** 10.2196/46021

**Published:** 2023-08-02

**Authors:** Tom Hähnel, Tim Feige, Julia Kunze, Andrea Epler, Anika Frank, Jonas Bendig, Nils Schnalke, Martin Wolz, Peter Themann, Björn Falkenburger

**Affiliations:** 1 Department of Neurology University Hospital and Faculty of Medicine Carl Gustav Carus Technische Universität Dresden Dresden Germany; 2 German Center for Neurodegenerative Diseases (DZNE) Dresden Germany; 3 Department of Neurology and Geriatrics Elblandklinikum Meißen Meißen Germany; 4 Department of Neurology Klinik am Tharandter Wald Halsbrücke Germany

**Keywords:** cognition, executive function, language function, mild cognitive impairment, Parkinson disease, Parkinson disease dementia, semantic clusters, semantic relatedness, verbal fluency tasks

## Abstract

**Background:**

Phonematic and semantic verbal fluency tasks (VFTs) are widely used to capture cognitive deficits in people with neurodegenerative diseases. Counting the total number of words produced within a given time frame constitutes the most commonly used analysis for VFTs. The analysis of semantic and phonematic word clusters can provide additional information about frontal and temporal cognitive functions. Traditionally, clusters in the semantic VFT are identified using fixed word lists, which need to be created manually, lack standardization, and are language specific. Furthermore, it is not possible to identify semantic clusters in the phonematic VFT using this technique.

**Objective:**

The objective of this study was to develop a method for the automated analysis of semantically related word clusters for semantic and phonematic VFTs. Furthermore, we aimed to explore the cognitive domains captured by this analysis for people with Parkinson disease (PD).

**Methods:**

People with PD performed tablet-based semantic (51/85, 60%) and phonematic (69/85, 81%) VFTs. For both tasks, semantic word clusters were determined using a semantic relatedness model based on a neural network trained on the Wikipedia (Wikimedia Foundation) text corpus. The cluster characteristics derived from this model were compared with those derived from traditional evaluation methods of VFTs and a set of neuropsychological parameters.

**Results:**

For the semantic VFT, the cluster characteristics obtained through automated analyses showed good correlations with the cluster characteristics obtained through the traditional method. Cluster characteristics from automated analyses of phonematic and semantic VFTs correlated with the overall cognitive function reported by the Montreal Cognitive Assessment, executive function reported by the Frontal Assessment Battery and the Trail Making Test, and language function reported by the Boston Naming Test.

**Conclusions:**

Our study demonstrated the feasibility of standardized automated cluster analyses of VFTs using semantic relatedness models. These models do not require manually creating and updating categorized word lists and, therefore, can be easily and objectively implemented in different languages, potentially allowing comparison of results across different languages. Furthermore, this method provides information about semantic clusters in phonematic VFTs, which cannot be obtained from traditional methods. Hence, this method could provide easily accessible digital biomarkers for executive and language functions in people with PD.

## Introduction

### Cognitive Deficits in People With Parkinson Disease

Parkinson disease (PD) is the fastest growing neurological disease and the second most common neurodegenerative disease [1]. Cognitive deficits are a frequent problem in people with PD. Approximately 10% to 20% of people with PD show a mild cognitive impairment [2], and approximately 46% of people with PD develop PD dementia (PDD) within 10 years after diagnosis [3]. PDD results in higher health-related costs and a reduced quality of life and, therefore, is of high importance for affected people and health care systems [2]. In addition, PDD constitutes 1 of the 4 milestones that occur, on average, 4 years prior to death and usher the terminal phase of the disease [4].

Cognitive decline in people with PD is characterized by deficits in attention, executive functions, visuospatial functions, memory, and language function [5]. Cognitive functions in people with PD are normally measured using paper-based neuropsychological tests [5,6]. These tests are time-consuming and require experienced raters.

### Clusters in the Verbal Fluency Task Transcript

Verbal fluency tasks (VFTs), by contrast, require less time and can report both executive and language functions [5,7]. There are 2 types of VFTs. In the semantic VFT, participants have to produce as many words as possible from 1 specific semantic category within 1 minute. The category “animal” is used most often. In the phonematic VFT, participants have to produce as many words as possible starting with a specific letter within 1 minute. Counting the total number of words produced by the person constitutes the most common analysis for both types of VFTs.

People generally do not produce these words in an evenly spaced temporal sequence but in clusters that often share semantic or phonematic similarities [8-15]. Traditionally, these clusters have been determined manually using 2 methods described by Troyer et al [9]. Words produced in the semantic VFT tend to form clusters of semantically related words. For example, a participant could start with a cluster of pets (dog and cat) and switch to a cluster of animals from Africa (elephant, giraffe, and lion). In the traditional analysis, the identification of these clusters and switches between clusters is based on predefined lists of, for example, animals (eg, a list of African animals and a list of pets). Words produced in the phonematic VFT have traditionally been analyzed using a set of phonematic rules [9]. One of the rules, for example, is to group words starting with the same 2 letters (eg, simple, simulate, and silly).

After the identification of word clusters, characteristics such as the mean cluster size and number of switches between clusters are calculated. Several studies suggested that the size of clusters is associated with language functions [10,14,16], whereas the number of switches is more strongly associated with executive functions [10,17]. Other authors, however, obtained conflicting results [12,17,18], and some studies found the cluster size and number of clusters to be highly correlated, which means that they might not represent independent parameters at all [7,17,19].

Analysis of word clusters in the VFT has been limited by several factors. First, the lists used to analyze the semantic VFT must be created manually, which entails subjectiveness. Second, they can exclusively be used for only 1 language. Third, simple lists may not capture all the individual associations that occur during testing. Fourth, the relatedness of consecutive words can only be classified dichotomously, that is, the word either belongs to the same cluster or not. Thus, it is not possible to quantify the semantic or phonematic “distance” of consecutive words.

In recent years, several approaches have been developed to overcome these disadvantages and allow for an automated, more objective, and quantitative analysis. In general, these approaches identify semantic clusters based on the semantic relatedness of words using mathematical models trained on a large text corpus [20-31].

In some approaches, semantic relatedness is directly estimated from structured knowledge sources such as ontologies or encyclopedias. For example, databases storing hierarchical relations between words have been used to estimate semantic relatedness [20]. Thus, an ontology where *cat* and *dog* are both elements of the parent group *carnivore* leads to a higher semantic relatedness between these animals than between *cat* and *cow.* Other models estimate semantic relatedness based on the link structure between web-based encyclopedia articles [32]. These models make explicit use of knowledge created by humans, but they require complex and highly structured training sets.

A more widely used approach for estimating semantic relatedness is the *latent space analysis*, which is based on the co-occurrence of words in training texts [21-23]. Thereby, 2 words are assumed to be semantically related if they co-occur with similar words in the training texts.

Finally, recent approaches also consider the position of words in relation to each other [24-29]; *Word2Vec*, for instance, uses a sliding window to estimate semantic relatedness by analyzing surrounding words [33]. In this approach, a neural network is trained to predict a word given its surrounding words (continuous bag-of-words method) or to predict the surrounding words given a centered word (skip-gram method). On the basis of this training, semantic relatedness can be estimated from the similarity of the learned context in which these words occur.

Most previous studies analyzed VFTs performed by people with mild cognitive impairment, Alzheimer disease [20,25,28,30], or psychiatric diseases [22,23,27,29]. For people with PD, there is only very limited evidence from 1 study [17]. In this study, Farzanfar et al [17] showed that applying a semantic relatedness model to semantic VFTs performed by people with PD is feasible. Here, executive function correlated with the number of cluster switches but not with the cluster size. Whether a semantic relatedness model can also be applied to phonematic VFTs performed by people with PD has not been explored yet.

### Aim of This Study

The aim of this study was to evaluate the feasibility of an automatic cluster analysis based on semantic relatedness in people with PD using voice recordings of semantic and phonematic VFTs. In addition, we aimed to validate the potential of the resulting cluster parameters as digital biomarkers for executive and language functions in people with PD. Finally, we provide our previously trained models for semantic relatedness in different languages to facilitate further research by others [34,35].

### Clinical Implications

Our study provides a tool for the automatic identification of semantically related word clusters in VFTs. VFTs are a widely used assessment for capturing cognitive functions in clinical practice and research. Although counting the total number of words produced within 1 minute constitutes the commonly used analysis for VFTs, further analysis of word clusters can provide additional information about executive and language functions. However, traditional methods of cluster identification lack standardization, require considerable manual work, and are language specific, thus limiting their applicability.

We show that cluster identification is possible using a model of semantic relatedness that overcomes these limitations. We prove that this automated approach provides valid digital biomarkers for executive and language functions.

By publishing our source code together with a readily trained model, we will allow other researchers to easily use this approach in their own studies. Thus, this study will allow further trials to capture more information about executive and language impairments without requiring additional time-consuming assessments. Furthermore, this will lead to more reliable and better comparable measurements of executive and language functions as cognitive outcomes in clinical trials. This approach is not limited to people with PD and can also be applied to people with other diseases with impaired executive or language function.

## Methods

### Recruitment

People with PD were recruited from 3 inpatient and outpatient movement disorder clinics in east Saxony, Germany, between May 2021 and August 2022. Participants with a clinically probable diagnosis of PD according to the current clinical diagnostic criteria [36], sufficient German language skills, and a Montreal Cognitive Assessment (MoCA) score >15 were included in the study. The severity of motor symptoms was assessed using the Movement Disorder Society-Sponsored Revision of the Unified Parkinson’s Disease Rating Scale (MDS-UPDRS) subscale III [37]. Levodopa equivalent doses were calculated using the recommended conversion factors [38].

### Ethics Approval

This study was approved by the institutional review board of Technische Universität Dresden, Germany (IRB00001473 and BO-EK-149032021). Written informed consent was obtained from all the participants before inclusion in the study.

### Phonematic and Semantic VFTs

Phonematic and semantic VFTs were performed without supervision using a self-developed app on an iPad 8 (Apple Inc) running iOS version 14. The semantic VFT was added later to the app, thus leading to fewer recordings for this task. For both VFTs, words with the same word stem, word repetitions, and proper names were not allowed. Instructions for the VFTs outlining these rules were presented to the participants before the test on the tablet. The phonematic VFT was performed first, and the semantic VFT was performed second. After reading the general instructions, the participant was requested to continue to the next page. At this time, the letter “S” (for the phonematic VFT) or the category “animals” (for the semantic VFT) was shown, and the voice was recorded for 60 seconds using the tablet’s internal microphone. Speech was detected and transcribed automatically using the Apple Speech Framework (Apple Inc) in iOS 14, which allows local speech processing on the device itself. The transcripts were checked manually by an investigator, and speech recognition errors were corrected. The transcripts were also checked for words that violated any of the aforementioned rules. Transcripts with >25% of violations were excluded from the analysis. In addition, recordings with no words spoken within the first 10 seconds were removed from the analysis because it was deemed unclear whether the person had understood the task.

### Speech Recognition Error Rate Calculation

The error rate of the automatically transcribed VFT recordings was measured as normalized Levenshtein distance. Therefore, we counted the numbers of insertions, deletions, and substitutions of words that would be required to change the automatically transcribed word list to the correct word list. This was done using the Python package *pylev* (version 1.4) [39]. Levenshtein distance was normalized by dividing it by the number of words in the correct word list.

### List-Based Clustering of the Semantic VFT

Traditional cluster analysis of the semantic VFT is based on fixed thematic lists of animals. These are based on shared features, such as geographical regions (eg, Africa), habitats (eg, water, farm, and pets), or species (eg, birds). To create these categorical lists, we translated the categories and animal lists used in the study by Troyer [11]. All animal words that were not covered by this translation were assigned to existing categories by the judgment of an investigator (TH), and additional categories were created as needed. Animal words were allowed to be part of multiple lists (eg, parrot is part of the lists “pet” and “bird”). The resulting categories and corresponding animal word lists can be found in Multimedia Appendix 1. Clusters were formed of consecutive words that occurred on at least 1 common list. The size of a cluster was calculated as the number of words within the cluster minus 1. The mean cluster size was obtained by also including clusters of single words. The number of switches was defined as the number of clusters, including clusters of single words, minus 1. To maintain consistency with the protocol of Troyer et al [9], rule violations were not excluded in the calculation of these cluster characteristics. The total word count comprised the number of words after removing rule violations.

### Rule-Based Clustering of the Phonematic VFT

Traditional cluster analysis of the phonematic VFT is based on fixed phonematic rules. Usually, four rules are used to identify words belonging to a cluster: (1) words starting with the same 2 letters (eg, summer and Sunday); (2) rhyming words (eg, sand and stand); (3) words differing only in 1 vowel sound (eg, sat and seat); and (4) homonyms, if indicated by the test person (eg, some and sum) [9]. Clusters were formed of consecutive words that fulfilled at least 1 common phonematic rule. The size of a cluster was calculated as the number of words within the cluster minus 1. The mean cluster size was obtained by also including clusters of single words. The number of switches was defined as the number of clusters, including clusters of single words, minus 1. To maintain consistency with the protocol of Troyer et al [9], rule violations were not excluded in the calculation of these cluster characteristics. The total word count comprised the number of words after removing rule violations.

### Semantic Relatedness Clustering

In addition to the traditional clustering methods, we implemented a semantic relatedness model based on a Word2Vec approach. In brief, this model is based on a neural network that depicts the semantic context of words in texts. To achieve this, the neural network is trained on a large text corpus in which the words surrounding each given word are analyzed. As a result, the semantic context of each word can be represented as a high-dimensional vector. The semantic relatedness (“distance”) of 2 words can be expressed as the cosine between 2 of these vectors.

The Word2Vec model was created and trained using the Python package g*ensim* version 4.0.1 [40] with Python 3.9.5 [41]. We used the freely available German Wikipedia (Wikimedia Foundation) corpus for model training [42]. To obtain optimal training results, 3 hyperparameters needed to be set: the dimensionality of the semantic relatedness space, window size for the surrounding words, and training algorithm. In addition, a fixed threshold for semantic relatedness needed to be set to define the word clusters. To find the optimal hyperparameter values, we performed a grid search using the following values: (1) dimensions: 200, 500, and 1000; (2) window size: 4 and 10; and (3) algorithm: continuous bag-of-words and skip-gram. To find the best semantic relatedness threshold, this parameter was varied between 0 and 1 with a step size of 0.01. We prevented overfitting by not training the hyperparameters directly on the word sequences obtained from the participants of this study. Instead, random pairs of animals were drawn from the animal category lists described earlier. We determined the set of hyperparameters that best detected whether both animals in a given pair shared a similar category list. For the comparison with the animal category lists, a semantic relatedness threshold of 0.40 performed the best in the approach described earlier and was used for the semantic VFT. For the phonematic VFT, the semantic relatedness threshold was set to a lower value (0.30) to allow for reasonably sensitive cluster identification. The hyperparameters identified using this approach for both VFTs are summarized in Table 1. Clusters were identified as follows: the words listed by a person were analyzed as a sequence of word pairs (word 1 and word 2, word 2 and word 3, ...). A cluster was defined as a sequence of word pairs in which each sequential word pair had a semantic relatedness greater than the thresholds stated earlier. The size of a cluster was calculated as the number of words within the cluster minus 1. The mean cluster size was obtained by also including clusters of single words. The number of switches was defined as the number of clusters, including clusters of single words, minus 1. To maintain consistency with the protocol of Troyer et al [9], rule violations were not excluded in the calculation of these cluster characteristics. The mean sequential semantic relatedness was determined by calculating the mean of the semantic relatedness of the sequence of all word pairs. The exact implementation of our semantic relatedness method and both traditional methods, including formulas, hyperparameters, and source code, can be obtained from our GitHub page [35]. Furthermore, the provided source code can be easily used to train models in other languages or based on other text corpora. In addition to the German model, we provide models pretrained on the English, Spanish, and French Wikipedia corpora using the same hyperparameters as those stated earlier [34,35].

**Table 1:** Hyperparameters used for training the semantic relatedness model and identifying semantically related clusters.

Parameters	Semantic VFT^a^	Phonematic VFT
Dimensions of semantic relatedness space	500	500
Word2vec window size	10	10
Word2vec algorithm	Skip-gram	Skip-gram
Semantic relatedness threshold	0.40	0.30

^a^VFT: verbal fluency task.

The listed parameters are the result of hyperparameter optimization, which is described in detail in this section. Different semantic relatedness thresholds were used for the semantic and phonematic VFTs. All other hyperparameters used for model training were identical between both VFTs.

### Paper-Based Neuropsychological Tests

The overall cognitive function of all the participants in this study was assessed using the MoCA [43]. In addition, the Frontal Assessment Battery (FAB) and Trail Making Test (TMT) B were used as measures of executive function [44,45]. The Boston Naming Test (BNT) [44] and Mehrfachwahl-Wortschatz-Intelligenztest B (MWT) [46] were performed to measure language function and crystallized intelligence. In the MWT, the participant has to distinguish existing words from fictive words in several word lists. The German versions of all the aforementioned tests were used.

### Statistical Analyses

The correlation of the MDS-UPDRS III item “Dysarthria” with the speech recognition error rate was calculated using Spearman rank correlation. All other correlations were calculated as Pearson correlations. For the comparison of the neuropsychological test results, a Mann-Whitney *U* test was performed because the neuropsychological test results were not normally distributed. Statistical tests were performed using Python 3.10.8 [41] with the *scipy* 1.9.3 package [47]. The network graph was created using the *networkx* 2.8.8 Python package [48]. For clarity, the following data are not shown in the network graph: correlations between 2 neuropsychological test results, neuropsychological test results with no correlation *with* the clustering characteristics, and Pearson correlation coefficients between 2 cluster characteristics.

## Results

### Patient Characteristics and Speech Recognition

**Figure 1:**
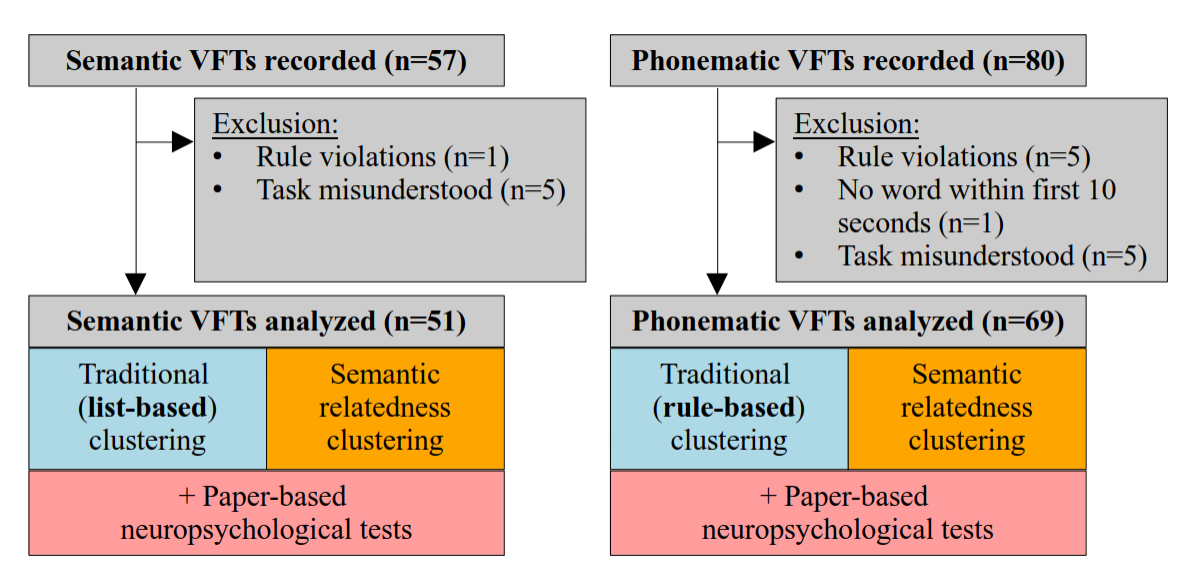
Block diagram of the study design. VFT: verbal fluency task.

In total, 137 recordings were obtained from 85 people with PD, specifically 80 recordings (94% of participants) for the phonematic VFT and 57 (67% of participants) for the semantic VFT. Of the 137 recordings, 6 (4.4%) recordings (phonematic: n=5, 6%; semantic: n=1, 2%) were excluded because the rules of the test were violated, 1 (1%) phonematic recording was removed because not a single word was spoken within the first 10 seconds of the task, and 5 (6%) phonematic and 5 (9%) semantic recordings were excluded because the participants misunderstood the task. This resulted in 69 (out of 80, 86%) phonematic VFT and 51 (out of 80, 89%) semantic VFT transcripts, which were used for traditional and semantical relatedness analyses (Figure 1).

Clinical characteristics of the patients are listed in Table 2. The recordings were transcribed using automatic speech recognition and checked manually for errors. The total error rate, calculated as normalized Levenshtein distance for both VFTs, was 61.8%. In detail, the semantic VFT showed a somewhat lower error rate (52%) than the phonematic VFT (69%), but this difference was not statistically significant (*P*=.15; Figure S1A in Multimedia Appendix 2). Furthermore, the error rate correlated significantly with the extent of dysarthria as reported by the corresponding MDS-UPDRS III item (*ρ=*0.26, *P*=.005; Figure S1B in Multimedia Appendix 2).

**Table 2:** Clinical characteristics of people with Parkinson disease included in the phonematic and semantic analyses.

Parameter			Phonematic VFT^a^ (n=69)	Semantic VFT (n=51)
Age (years), mean (SD)			61.2 (13.1)	60.4 (12.3)
**Sex, n (%)**				
		Female	27 (39)	16 (31)
		Male	42 (61)	35 (69)
**Hoehn and Yahr ON^b^, n (%)**				
		Mild (0-2)	52 (75)	44 (86)
		Moderate (2.5-3)	14 (20)	6 (12)
		Severe (4-5)	3 (4)	1 (2)
Disease duration (years), mean (SD)			7.4 (5.1)	5.9 (4.0)
**Subtype, n (%)**				
		Tremor dominant	13 (19)	11 (22)
		Akinetic rigid	28 (41)	19 (37)
		Mixed	28 (41)	21 (41)
LEDD^c^, mean (SD)			692 (356)	670 (349)
MDS-UPDRS^d^ III, mean (SD)			21 (12)	19 (10)
MoCA^e^ score, mean (SD)			26.5 (2.6)	26.7 (3.0)
**DBS^f^, n (%)**				
	Yes		7 (10)	4 (8)
	No		62 (90)	47 (92)

^a^VFT: verbal fluency task.

^b^People with Parkinson disease can be examined in an ON or OFF state. ON refers to the typical functional state when patients are receiving medication and have a good response.

^c^LEDD: levodopa equivalent daily dose.

^d^MDS-UPDRS: Movement Disorder Society‐Sponsored Revision of the Unified Parkinson's Disease Rating Scale.

^e^MoCA: Montreal Cognitive Assessment.

^f^DBS: deep brain stimulation.

### Traditional Clusters and Semantically Related Clusters

Cluster characteristics were analyzed for both phonematic and semantic VFTs using (1) traditional clustering methods and (2) the novel semantic relatedness method.

**Figure 2:**
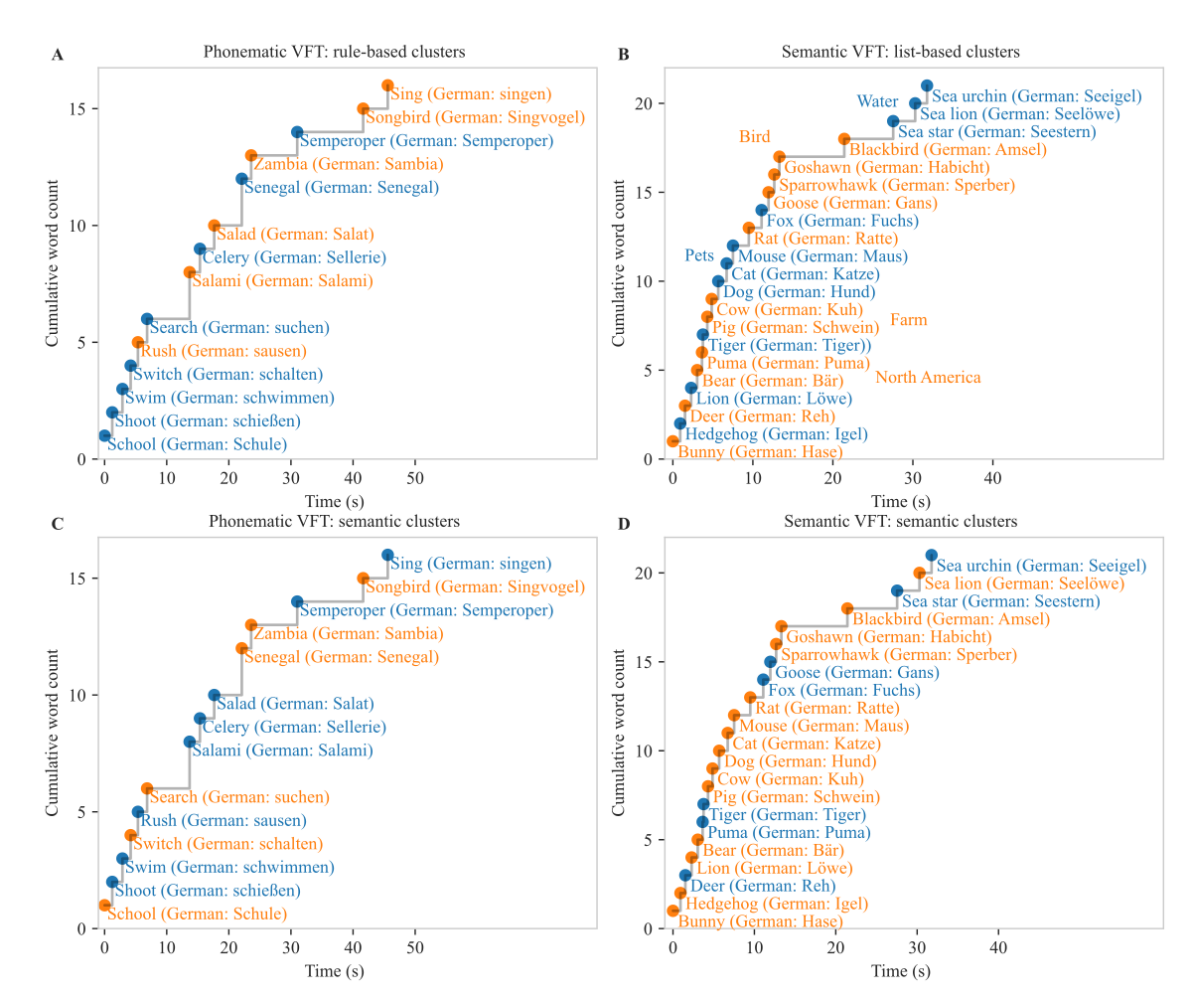
Phonematic and semantic clustering examples. Cluster examples for the traditional rule-based (A) and list-based (B) technique and the semantic relatedness technique (C and D). Words belonging to the same cluster are displayed in the same color (in blue or orange). For the list-based clustering (B), the common lists for clusters with >1 word is displayed next to each word pair. VFT: verbal fluency task.

For the phonematic VFT, the traditional cluster analysis is based on phonematic rules, as described in the *Methods* section. In our data, most of the phonematic word pairs (297 word pairs) were identified as clusters because the words shared the same first 2 letters. Only a few clusters were identified by applying the remaining phonematic rules: 6 word pairs were identified as clusters because the words rhymed, 1 word pair was identified as a cluster because the words differed only in 1 vowel, and no homonyms were found. An example of rule-based phonematic clusters is shown in Figure 2A.

**Figure 3:**
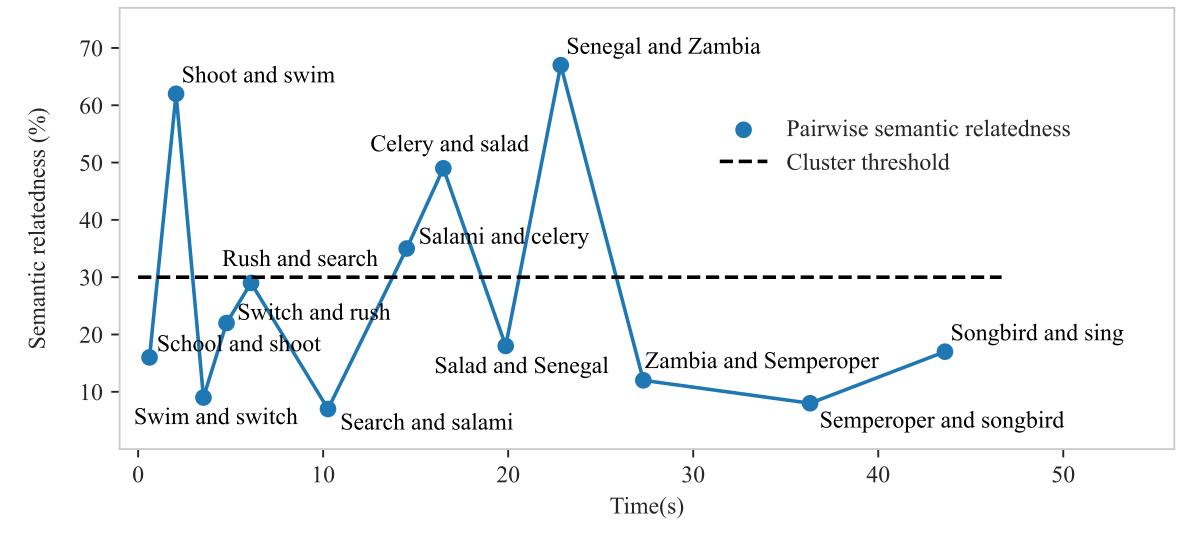
Identification of clusters by calculating the pairwise semantic relatedness. The figure depicts the pairwise semantic relatedness of all sequential word pairs from the phonematic verbal fluency task (VFT) shown in Figure 2C. Words with a pairwise semantic relatedness above the threshold form clusters.

**Figure 4:**
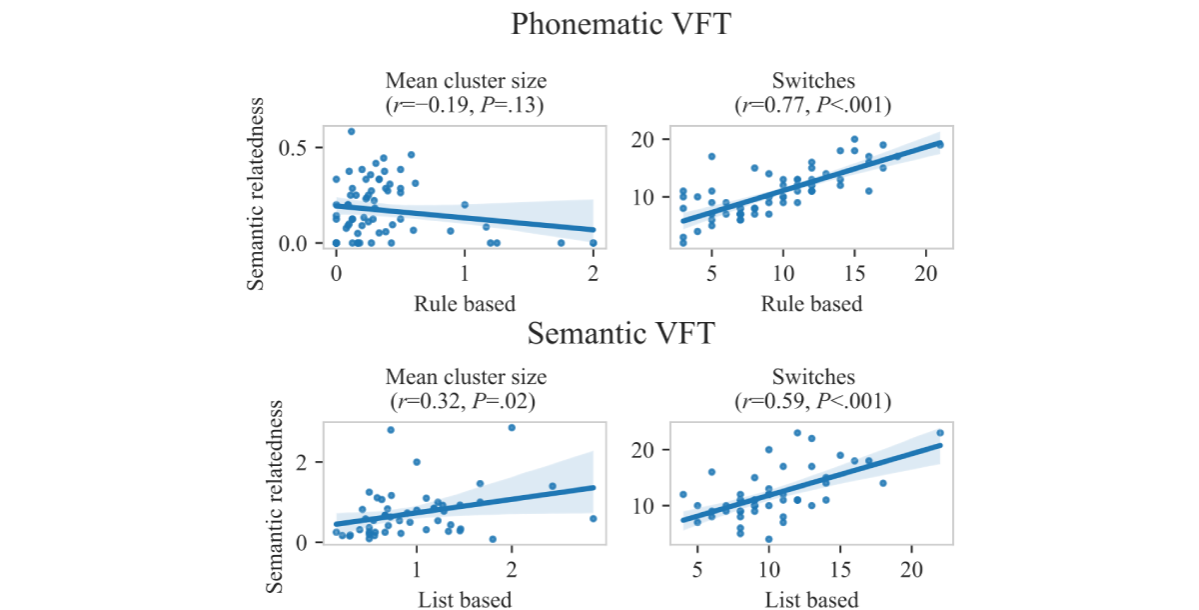
Correlation of traditional rule- and list-based and semantic relatedness cluster characteristics. Correlations of the semantic-related cluster characteristics (y-axis) with the traditional rule- and list-based cluster characteristics (x-axis) for the phonematic verbal fluency task (VFT; top row) and the semantic VFT (bottom row). Pearson correlation coefficients and corresponding *P* values are shown.

In contrast to these phonematic rules, the semantic relatedness method identifies clusters based on a model that can quantify the relatedness of word pairs (Figure 3). The semantic relatedness model was trained on the German Wikipedia corpus. On the basis of this large training data set, this method can identify entirely different clusters from those identified through the rule-based system, for example, the sequence salad, celery, and salami (German: *salat*, *sellerie*, and *salami*) or Zambia and Senegal (German: *Sambia* and *Senegal*), in which words did not share phonematic similarities (Figure 2C). Compared with the traditional rule-based clustering method, in the semantic relatedness method, the clusters had a smaller size, and switches between clusters occurred slightly more often (Table 3). Nonetheless, the number of switches obtained through both methods correlated strongly (*r*=0.77; *P*<.001), whereas the mean cluster size did not correlate between the clustering methods (*P*=.13; Figure 4), potentially because these clusters were construed differently.

For the semantic VFT, the traditional clustering method is based on lists of animals with different themes (eg, farm animals or birds). Words are recognized as a cluster if they are found on at least 1 common list. An example of such list-based clusters is shown in Figure 2B. The clusters in the semantic VFT identified through the list-based method were in general comparable with those identified through the semantic relatedness approach (Figure 2D). This is consistent with the fact that the clusters were generated in a more similar way for the semantic VFT than for the phonematic VFT. However, some additional clusters were detected through the semantic relatedness method. For instance, the cluster bunny and hedgehog may be based on a familiar German fairy tale, and the cluster fox and goose may be based on a common German nursery rhyme.

As for the phonematic VFT, switches between clusters occurred slightly more often with the semantic relatedness method than with the traditional list-based clustering method, and the clusters identified through the semantic relatedness method were slightly smaller than those identified through the traditional list-based clustering method (Table 3). The numbers of switches obtained using the 2 methods correlated significantly (*r*=0.59; *P*<.001), as did the cluster sizes (*r*=0.32; *P*=.02; Figure 4). The strength of the correlation observed in our work is comparable with that observed in a recent study that analyzed traditional and semantic clusters obtained from the semantic VFT performed by people with PD [17].

**Table 3:** Characteristics of the traditional (rule and list based) and semantic relatedness clusters.

	Phonematic VFT^a^		Semantic VFT	
	Rule based	Semantic relatedness	List based	Semantic relatedness
Total word count, mean (SD)	12.3 (4.6)	12.3 (4.6)	19.6 (5.7)	19.6 (5.7)
Mean cluster size, mean (SD)	0.4 (0.4)	0.2 (0.1)	0.9 (0.6)	0.7 (0.6)
Switches, mean (SD)	9.5 (4.1)	10.7 (4.0)	10.3 (3.5)	12.1 (4.4)
Mean sequential semantic relatedness (%), mean (SD)	N/A^b^	18.9 (4.4)	N/A	36.8 (5.3)

^a^VFT: verbal fluency task.

^b^N/A: not applicable.

Next, we investigated the effect of sex, age, and disease duration on clustering. Performing sex-specific subgroup analysis revealed no differences between male and female patients except for a slightly higher number of switches in female patients (10.9 vs 8.7; Tables S1 and S2 in Multimedia Appendix 2) in the semantic relatedness analysis of the phonematic VFT. In general, younger patients produced more total words (21.4 vs 17.5) than older patients in the semantic VFT. There were no differences in other cluster characteristics or the phonematic VFT (Tables S3 and S4 in Multimedia Appendix 2). Longer disease duration was associated with slightly more switches between semantically related clusters for the phonematic VFT (11.6 vs 9.6), whereas no difference was observed in other cluster characteristics or the phonematic VFT (Tables S5 and S6 in Multimedia Appendix 2).

Comparing the phonematic and semantic VFTs, we obtained higher total word counts (19.6 vs 12.3; *P*<.001) and larger clusters for the semantic VFT, which is consistent with previous research [49]. The semantic relatedness approach showed a much higher mean sequential relatedness between sequential words for the semantic VFT than for the phonematic VFT (36.8% vs 18.9%; *P*<.001; Table 3).

When we compared the cluster characteristics of the phonematic VFT and semantic VFT for each patient, we observed strong correlations independent of the method used for clustering (Figure S2 in Multimedia Appendix 2). Specifically, we found positive correlations between the total word count and the number of switches. A higher number of switches was associated with a lower mean cluster size, except for the semantic relatedness clusters of the phonematic VFT. Moreover, a higher mean sequential relatedness was associated with larger clusters in the semantic relatedness method, consistent with recent publications on this matter [17,19].

In summary, our semantic relatedness method produced meaningful results consistent with previous work by others. Therefore, we trained the semantic relatedness model on the English, French, and Spanish Wikipedia word corpora (see the *Methods* section) [34,35]. Examples of semantic relatedness clusters in these languages are presented in Figure S3 to S8 in Multimedia Appendix 2.

### Correlations With Neuropsychological Tests of Executive and Language Functions

To investigate which cognitive domains are captured by the above-described cluster characteristics, we compared the obtained cluster characteristics with the results of paper-based cognitive tests. In general, the VFT can be seen as a measure of executive and language functions; specifically, the number of switches is considered a measure of executive function, and the cluster size is considered a measure of language function [9,10]. To assess the executive domains in more detail, we used the FAB and TMT B. A lower TMT B score indicates a better result. To assess language function in a standardized fashion, we performed the MWT and BNT. Overall cognition was measured using the MoCA. Correlations between clustering characteristics and neuropsychological tests are shown in Table 4 for the phonematic VFT and in Table 5 for the semantic VFT.

**Figure 5:**
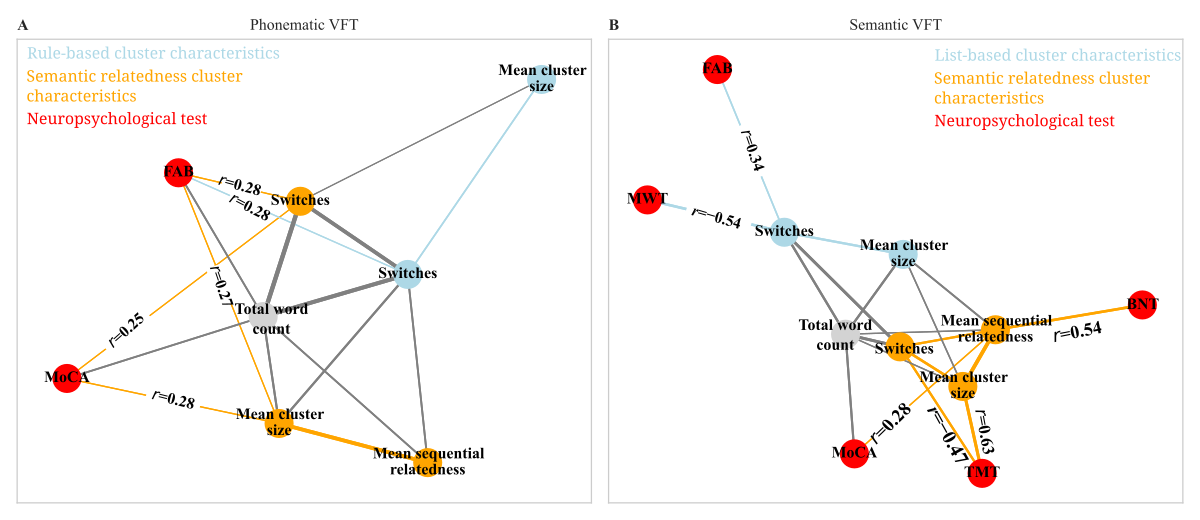
Network graph of Pearson correlations between clustering characteristics and neuropsychological test results. Significant (*P*<.05) correlations for the (A) phonematic and (B) semantic verbal fluency tasks (VFTs) are shown as a network graph. The thickness of the connections and the distance between parameters indicate the magnitude of the correlation (thicker lines and shorter distances indicate stronger correlations). The Pearson correlation coefficients are shown for correlations between clustering characteristics and neuropsychological test results. BNT: Boston Naming Test; FAB: Frontal Assessment Battery; MoCA: Montreal Cognitive Assessment; MWT: Mehrfachwahl-Wortschatz-Intelligenztest; TMT: Trail Making Test.

The most important readout of the VFT is the total word count. It correlated with the overall cognitive performance as measured by the MoCA for both the phonematic VFT (*r*=0.38; *P*=.002) and semantic VFT (*r*=0.45; *P*=.001). The MoCA also correlated significantly with cluster characteristics for both types of VFT obtained through the semantic relatedness method. Specifically, a higher MoCA score was associated with a higher mean sequential relatedness in the semantic VFT (*r*=0.28; *P*=.04), a higher mean cluster size (*r*=0.28; *P*=.02) in the phonematic VFT, and a higher number of switches (*r*=0.25; *P*=.04) in the phonematic VFT (Figure 5; Tables 4 and 5). Interestingly, no significant correlations with the MoCA were found for the cluster characteristics obtained through traditional clustering methods (Tables 4 and 5).

With respect to executive functions, the FAB score correlated significantly with the total word count (*r*=0.38; *P*=.005) and number of switches in the phonematic VFT obtained through the traditional rule-based clustering method (*r*=0.28; *P*=.04) and semantic relatedness method (*r*=0.28; *P*=.04). Larger clusters obtained from the semantic relatedness method were associated with a higher FAB for the phonematic VFT (*r*=0.27; *P*=.05). Regarding the semantic VFT, a higher number of switches obtained through the traditional method was associated with a higher FAB score (*r*=0.34; *P*=.04). Taken together, the FAB score thus correlated more strongly with the results of the phonematic VFT than with the results of the semantic VFT. This is consistent with previous findings by other studies [50,51]. Better TMT B results were associated with smaller clusters (*r*=0.63; *P*=.006) and a higher number of switches (*r*=−0.47; *P*=.05) for the semantic VFT as obtained through the semantic relatedness clustering method. The different correlations of FAB and TMT B demonstrate that executive function is not a homogeneous concept and support using different assessment methods. Collectively, these findings demonstrate that the semantic relatedness method can reproduce the association of VFT cluster characteristics with measures of executive function.

With respect to language function, interestingly, we observed no correlations of BNT and MWT with the clustering characteristics of the phonematic VFT. As for the semantic VFT, we found a lower number of switches to be associated with a higher MWT score (*r*=−0.54; *P*=.02) in the traditional clustering method. BNT scores correlated with the mean sequential relatedness of the semantic VFT in the semantic relatedness method (*r*=0.54, *P*=.02). These findings are consistent with the idea that clustering in VFTs is associated with language function [9,10] and demonstrate that the semantic relatedness method can reproduce associations of VFT cluster characteristics with language function.

**Table 4:** Correlations of the phonematic VFT^a^ cluster characteristics with neuropsychological test results.

		Overall cognition	Executive function			Language function	
		MoCA^b^	FAB^c^	TMT B^d^	BNT^e^		MWT^f^
**Total VFT word count**							
	*r*	*0.38* ^g^	*0.38*	−0.03	0.10		0.32
	*P* value	*.002*	*.005*	.89	.63		.12
**Mean cluster size (rule based)**							
	*r*	0.16	0.05	0.00	0.25		0.12
	*P* value	.20	.74	.99	.23		.58
**Switches (rule based)**							
	*r*	0.21	*0.28*	−0.06	0.06		0.30
	*P* value	.09	*.04*	.77	.78		.15
**Mean cluster size (semantic relatedness)**							
	*r*	*0.28*	*0.27*	−0.18	0.07		−0.04
	*P* value	*.02*	*.047*	.40	.72		.86
**Switches (semantic relatedness)**							
	*r*	*0.25*	*0.28*	0.03	0.15		0.35
	*P* value	*.04*	*.04*	.88	.47		.09
**Mean sequential relatedness (semantic relatedness)**							
	*r*	0.00	0.14	−0.15	−0.08		−0.12
	*P* value	.97	.32	.47	.71		.58

^a^VFT: verbal fluency task.

^b^MoCA: Montreal Cognitive Assessment.

^c^FAB: Frontal Assessment Battery.

^d^TMT B: Trail Making Test B.

^e^BNT: Boston Naming Test.

^f^MWT: Mehrfachwahl-Wortschatz-Intelligenztest.

^g^Significant values are in italics.

**Table 5:** Correlations of the semantic VFT^a^ cluster characteristics with neuropsychological test results.

		Overall cognition	Executive function		Language function	
		MoCA^b^	FAB^c^	TMT B^d^	BNT^e^	MWT^f^
**Total VFT word count**						
	*r*	*0.45* ^g^	0.24	−0.01	0.37	0.06
	*P* value	*.001*	.16	.96	.13	.80
**Mean cluster size (list based)**						
	*r*	0.22	−0.16	0.20	0.37	0.34
	*P* value	.12	.34	.42	.13	.17
**Switches (list based)**						
	*r*	0.15	*0.34*	−0.27	−0.12	−*0.54*
	*P* value	.30	*.04*	.28	.63	*.02*
**Mean cluster size (semantic relatedness)**						
	*r*	0.19	0.10	*0.63*	0.30	0.22
	*P* value	.18	.57	*.006*	.23	.39
**Switches (semantic relatedness)**						
	*r*	0.20	0.16	−*0.47*	0.01	−0.23
	*P* value	.16	.34	*.050*	.97	.35
**Mean sequential relatedness (semantic relatedness)**						
	*r*	*0.28*	0.07	0.45	*0.54*	0.25
	*P* value	*.045*	.69	.06	*.02*	.32

^a^VFT: verbal fluency task.

^b^MoCA: Montreal Cognitive Assessment.

^c^FAB: Frontal Assessment Battery.

^d^TMT B: Trail Making Test B.

^e^BNT: Boston Naming Test.

^f^MWT: Mehrfachwahl-Wortschatz-Intelligenztest.

^g^Significant values are in italics.

## Discussion

### Principal Findings

In this study, we present an automated approach to identify semantically related clusters in VFT transcripts. Speech recordings of semantic and phonematic VFTs were generated by people with PD without supervision using a tablet computer. The obtained cluster characteristics correlated with overall cognitive, executive, and language functions. Moreover, the cluster characteristics provided additional information compared with the total word count alone.

### Automatic Speech Recognition

The approach presented here allows for the automated execution and analysis of semantic and phonematic VFTs. By using a standard tablet computer and its integrated microphone, the test can be performed anywhere without the need for an experienced rater, making it a promising digital biomarker for the smartphone-based or tablet-based home monitoring of cognitive functioning.

However, the occurrence of a high percentage of speech recognition errors in automatic transcription for people with PD still limits the feasibility of completely automating this process for participants with dysarthria, consistent with previous results [52]. Advances in speech recognition technologies may help overcome this restriction in the future. The speech recognition error rate may already be lower for other languages and more advanced speech recognition algorithms [52].

### Advantages of the Semantic Relatedness Method

In contrast to traditional list-based and rule-based approaches, we used a mathematical model based on the semantic relatedness of words in a large text corpus to identify clusters and calculate the semantic relatedness between words. This demonstrated that the semantic relatedness model is different from and has advantages over the traditional approaches. First, this model allows for an exact and quantitative measurement of the semantic relatedness between 2 words. This is different from traditional methods, which only allow a dichotomous distinction, that is, whether words form a cluster or not.

Second, the estimation of semantic relatedness solely relies on the presence of words in the text corpus that was used for training the model. Thus, the detected clusters do not rely on the subjective decisions of the raters who manually created the word lists. For instance, we consider the clustering of words that occur together in fairy tales or nursery rhymes appropriate. In addition, we demonstrated that the semantic relatedness model can capture more complex relationships between words that go beyond simple lists of characteristics such as geographical regions or simple phonematic rules.

Within our German cohort, we found only a minimal number of rhymes and vowel-only differences and no homonyms for the phonematic VFT. This suggests language-specific differences in rule-based clusters, which limit their usability in an international research context. This limitation does not apply to the semantic relatedness model used in this study. In our view, this method might yield results that can be easily generalized to different languages. The strong correlation of the cluster characteristics of the phonematic VFT as determined by the semantic relatedness model with MoCA and FAB scores further substantiates the validity of this approach (Figure 5; Table 4). In addition, the semantic relatedness method allows for a comparison between the cluster characteristics of the semantic VFT and the cluster characteristics of the phonematic VFT.

### Advantages of the Automated Analysis

Using a semantic relatedness model as described above allows for the automation of cluster analysis in VFTs, which results in further advantages. Traditional list-based clustering requires a significant amount of manual work to create the animal lists and update them with new animals listed by the patients. If patients are to be tested again, a different category must be used for the modified test, and the 2 sets of lists might yield differing results. With the semantic relatedness approach, a modified VFT using a different category (eg, fruits instead of animals) can be analyzed using the same method without the need for extensive testing of the new word lists.

The traditional rule-based approach relies on manual work. The detection of homonyms depends on the meaning of the words, and the detection of rhymes depends on the pronunciation and not the spelling of the words. Both features would require more complex approaches, that is, databases identifying the meaning of words and algorithms incorporating the pronunciation of words. Such an automated phonetic analysis has been described, but it resulted in large differences between automated and manual cluster identification [53]. This manual work is not required when using a semantic relatedness model as described here.

As described earlier, the semantic relatedness model shows advantages when applied to different languages. Traditional list-based clustering requires the animal lists to be translated and adapted to the local and cultural circumstances. By contrast, the semantic relatedness model can be easily adapted using a freely available text corpus, such as Wikipedia in a different language. No specific adaptations or list translations need to be performed manually because all language-specific adaptations are already integrated into the text corpus used for training the model.

To further facilitate the use of the semantic relatedness method for VFT analysis, we publish with this manuscript pretrained models for the English, German, French, and Spanish languages, which are based on the corresponding language-specific Wikipedia corpora [34]. In addition, we provide a software to train the model, which will allow other researchers to apply the model to different corpora and new languages [35].

Despite these advantages, our approach also has several limitations. Most of the texts used for training the model are written text and not spoken language, some of which are written in a scientific style. Thus, a corpus of more common texts such as books or interviews may be more appropriate. Although the Wikipedia corpus is available in many languages, not all versions are as extensive as the German and English versions, which could potentially result in less accurate models. In this case, the corpus could be supplemented with books, newspaper articles, or other types of texts.

### Correlations of Cluster Characteristics With Neuropsychological Parameters

We used the traditional clustering methods to identify hyperparameters for the automated semantic relatedness method that provided a good correlation with the traditional method for the semantic VFT. For the phonematic VFT, the correlation between the automated semantic relatedness method and the traditional manual method was weaker. This can be explained by the different constructs used for phonematic rules and semantic relatedness.

We observed a correlation between executive functions and cluster characteristics, specifically the number of switches in the semantic and phonematic VFTs, which is consistent with previous data [13,17] and the concept that switching in VFTs reflects executive functioning [9,10]. We were able to replicate these findings for both semantic and phonematic VFTs in the semantic relatedness clustering method. Although semantic relatedness reflects a different construct compared with traditional rule-based clustering, the number of switches between semantically related clusters in the phonematic VFT also showed significant correlations with executive function as reported by the FAB. Regarding the language function, our results do not support the idea of cluster sizes as a marker of language function [9,10]. Similarly conflicting results were also reported by other researchers, and these showed either no correlations of clustering characteristics with language function or correlations of the number of switches with language function [17,18]. The heterogeneity of these results may be caused by the subjectiveness of the animal lists required for traditional clustering and by the correlation of the mean cluster size with the number of switches, as observed in our data and described elsewhere [7,17,19]. By applying the semantic relatedness method, we were able to observe that a higher mean sequential relatedness is associated with a higher BNT score. This shows that the cluster characteristics obtained through the semantic relatedness method yield additional information about language function that cannot be inferred from the total word count or from the traditional clustering method.

Because the phonematic and semantic VFTs were conducted in the same order in all patients, we cannot rule out a negative bias toward the second task caused by fatigue. We assume that the impact of not randomizing the order of the VFTs is limited because the VFT is a very short assessment taking only 1 minute to complete.

Overall, our semantic relatedness clustering method when applied to the semantic VFT yielded results comparable with those published in a recent study [17], highlighting a robust correlation with executive function in people with PD. Our study is the first to investigate semantically related clusters for the phonematic VFT in people with PD. In this study, we showed for the first time that the semantic relatedness method can also be applied to the phonematic VFT in people with PD and that the resulting clustering characteristics are a robust marker of executive function.

### Conclusions

In summary, our work demonstrates the feasibility of a standardized cluster analysis of semantic and phonematic VFT transcripts using a semantic relatedness model. This model overcomes numerous disadvantages of traditional clustering methods, allows for the automation of cluster identification, and shows strong correlations with executive functions. The presented automated approach enables a more objective identification of semantic clusters in different languages: going forward, it could help overcome the heterogeneity of previously published studies in this field. Longitudinal trials are required to determine whether cluster characteristics are associated with differences in cognitive decline or disease progression. In the future, this automated semantic relatedness method could provide easily accessible digital biomarkers for executive function in PD.

## Appendix

Multimedia Appendix 1 Animal lists used for traditional clustering.

Multimedia Appendix 2 Additional figures for speech recognition error rates and correlations between cluster characteristics and subgroup analyses (age, sex, and disease duration). Furthermore, we have presented semantic relatedness cluster examples for the languages English, Spanish, and French based on the corresponding language-specific Wikipedia corpuses.

## Abbreviations

BNT: Boston Naming Test
FAB: Frontal Assessment Battery
MDS-UPDRS: Movement Disorder Society-Sponsored Revision of the Unified Parkinson’s Disease Rating Scale.
MoCA: Montreal Cognitive Assessment
MWT: Mehrfachwahl-Wortschatz-Intelligenztest
PD: Parkinson disease
PDD: Parkinson disease dementia
TMT: Trail Making Test
VFT: verbal fluency task
