# Supplementary material for: A Semantic Relatedness Model for the Automatic Cluster Analysis of Phonematic and Semantic Verbal Fluency Tasks Performed by People With Parkinson Disease: Prospective Multicenter Study
Source: JMIR Neurotechnol. 2023 Aug 2;2:e46021. doi: 10.2196/46021 (PMC12671300; doi:10.2196/46021)
Supplement: Multimedia Appendix 2 [file neuro_v2i1e46021_app2.doc]

Multimedia Appendix

# Automatic Cluster Analysis using a Semantic Relatedness Model for the Phonematic and Semantic Verbal Fluency Task in Parkinson's Disease: Results from a Prospective Multicenter Study

**Tom Hähnel^1^, Tim Feige^2^, Julia Kunze^1^, Andrea Epler^1^, Anika Frank^1,2^, Jonas Bendig^1^, Nils Schnalke^1,2^, Martin Wolz^3^, Peter Themann^4^, Björn Falkenburger^1,2^**

1) Department of Neurology, University Hospital and Faculty of Medicine Carl Gustav Carus, TU Dresden, Dresden, Germany

2) German Center for Neurodegenerative Diseases (DZNE), Dresden, Germany

3) Department for Neurology and Geriatrics, Elblandklinikum Meißen, Meißen, Germany

4) Department for Neurology, Klinik am Tharandter Wald, Halsbrücke, Germany

Corresponding author:

Dr. med. Tom Hähnel

Department of Neurology

University Hospital Carl Gustav Carus,

Technische Universität Dresden

Fetscherstrasse 74

01307, Dresden, Germany

Email: tom.haehnel@uniklinikum-dresden.de

**
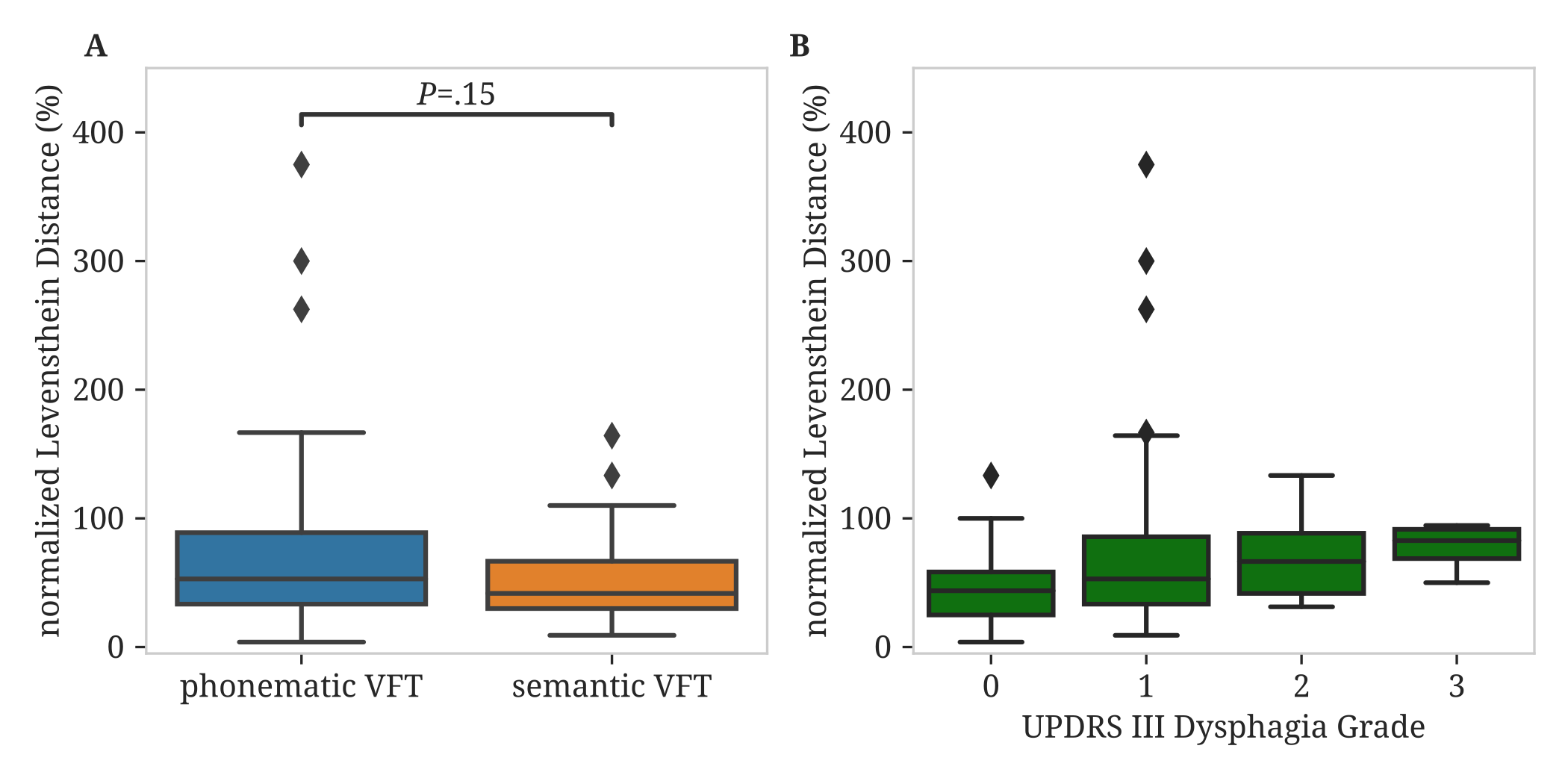
**

**Figure S1: Speech recognition error rates.**

The figure depicts the error rate of the automatic speech recognition software used for transcribing the audio files. The error rate is measured as normalized Levensthein Distance. **A**: Comparison of error rates for the phonematic and semantic VFT. **B**: Comparison of error rates for both semantic and phonematic recordings grouped by dysarthria severity as reported by the MDS UPDRS Part III dysarthria item (0: none, 1: slight, 2: mild, 3: moderate).

**
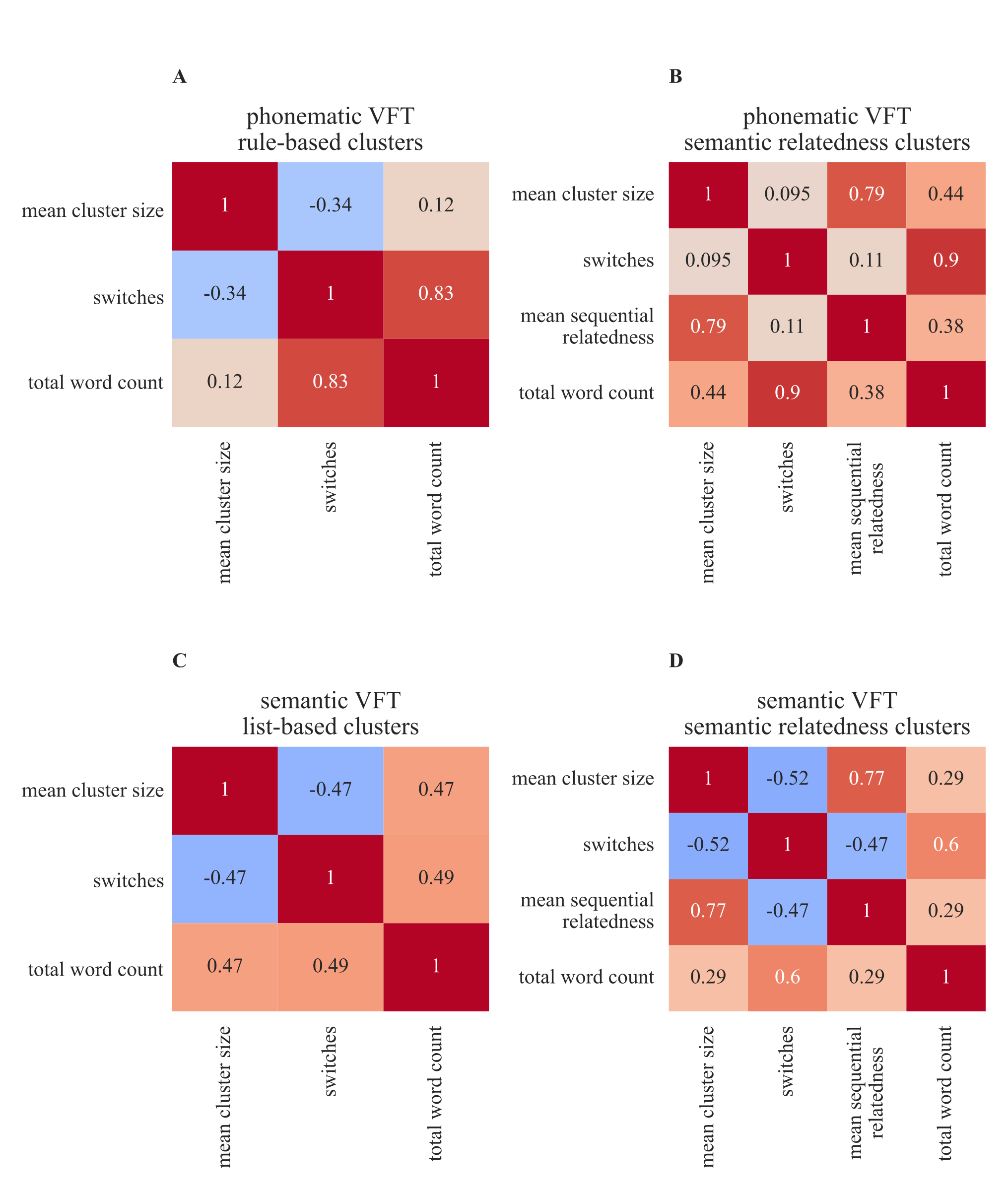
**

**Figure S2: Heatmap depicting the Pearson correlations between clustering characteristics**

The figure shows the strength of the Pearson correlations between clustering characteristics (blue = negative correlation, white = no correlation, red = positive correlation) for both types of VFTs and clustering methods. **A**: Correlations between rule-based clustering characteristics for the phonematic VFT. **B**: Correlations between semantic relatedness cluster characteristics for the phonematic VFT. **C**: Correlations between list-based clustering characteristics for the semantic VFT. **D**: Correlations between semantic relatedness cluster characteristics for the semantic VFT.

The semantic relatedness models used for the following diagrams can be obtained from the corresponding GitHub repository (https://github.com/t-haehnel/VFTModels, accessed Dec 12, 2022).

**
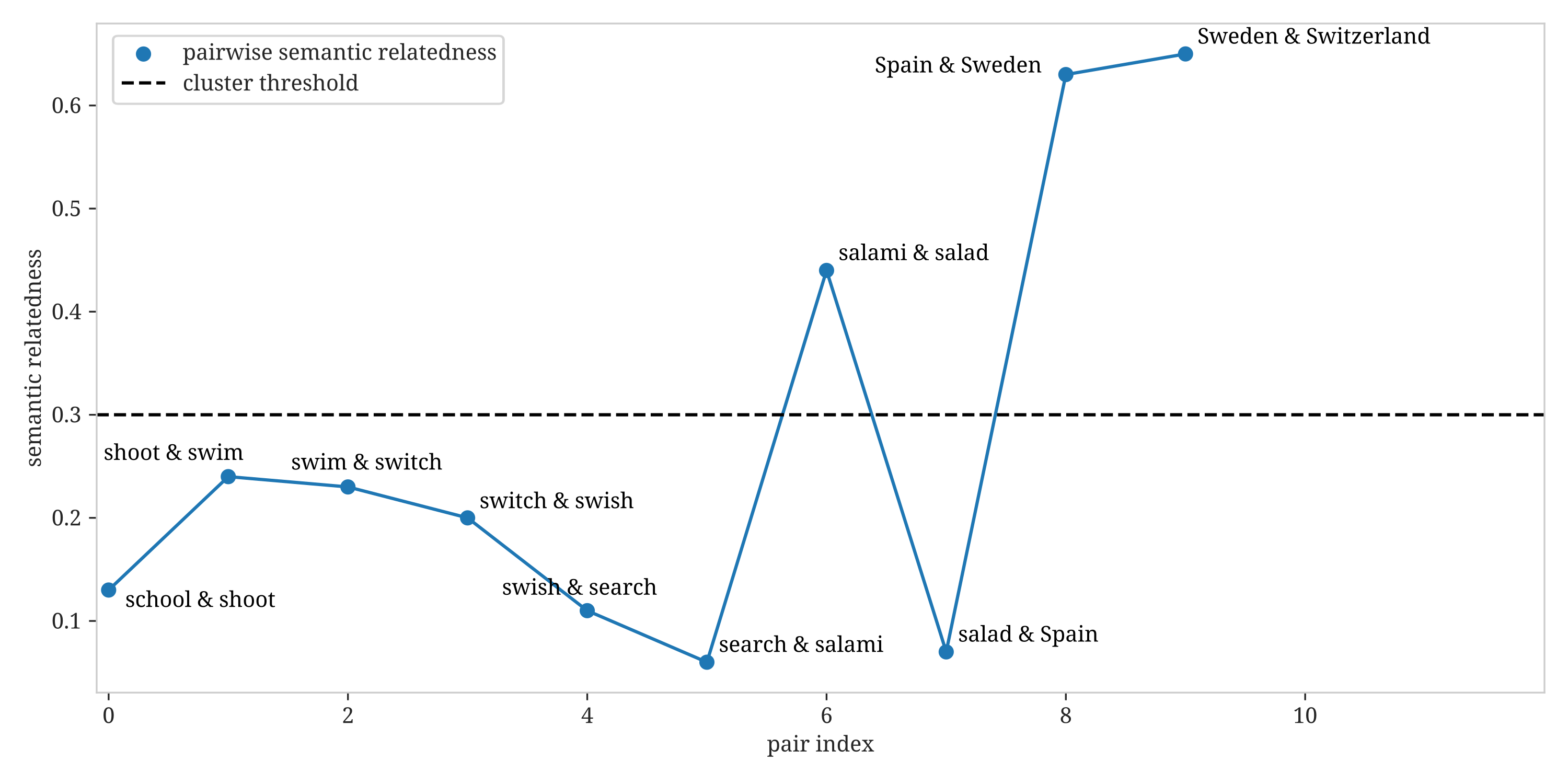
**

**Figure S3: Pairwise similarity of phonematic VFT (English).**

The figure depicts the pairwise semantic relatedness of all sequential word pairs from a phonematic VFT in English. Each series of words which shows a pairwise semantic relatedness above the fixed threshold (dashed line) forms a cluster.

**
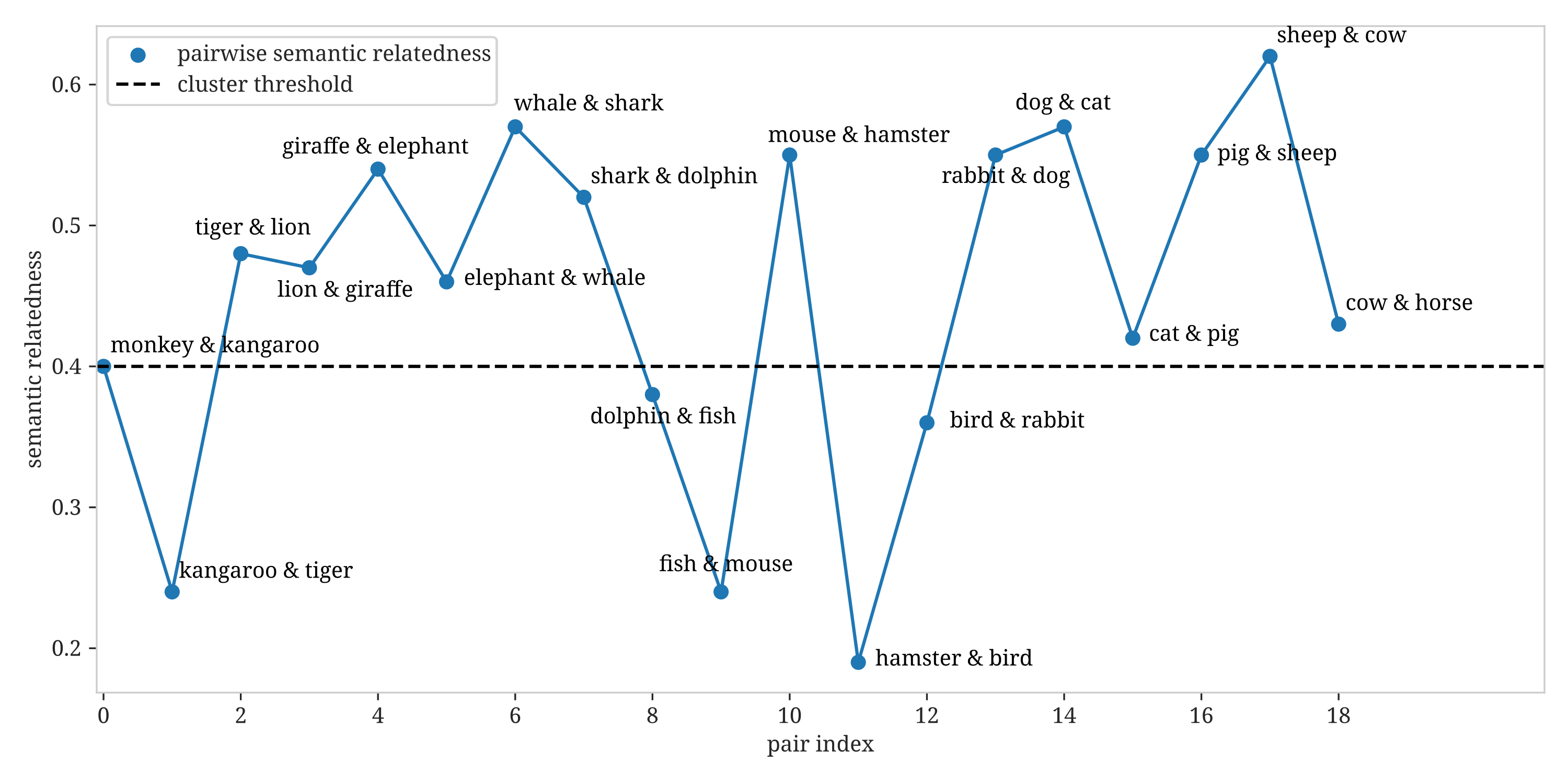
**

**Figure S4: Pairwise similarity of semantic VFT (English)**

The figure depicts the pairwise semantic relatedness of all sequential word pairs from a semantic VFT in English. Each series of words which shows a pairwise semantic relatedness above the fixed threshold (dashed line) forms a cluster.

**
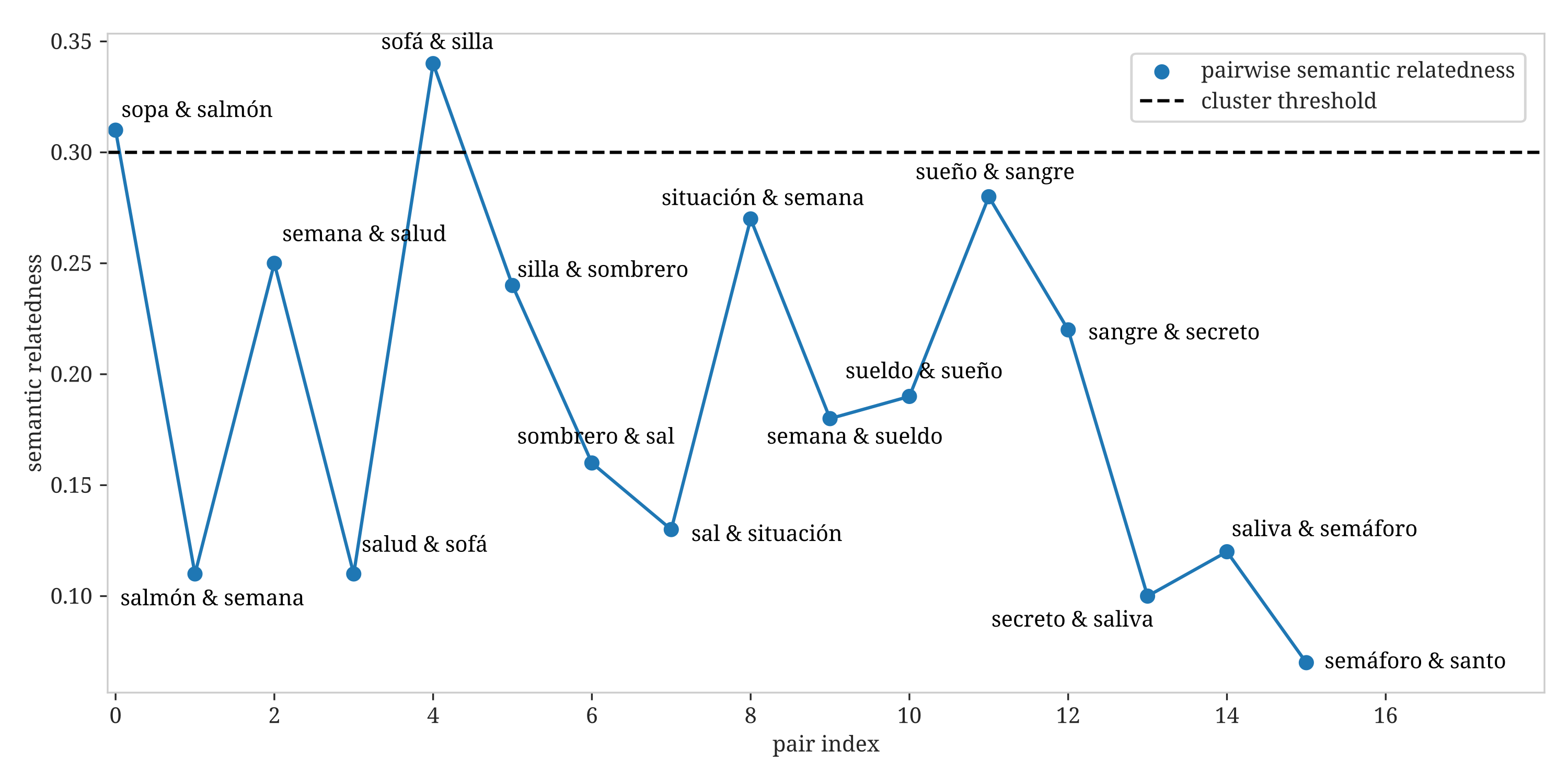
**

**Figure S5: Pairwise similarity of phonematic VFT (Spanish).**

The figure depicts the pairwise semantic relatedness of all sequential word pairs from a phonematic VFT in Spanish. Each series of words which shows a pairwise semantic relatedness above the fixed threshold (dashed line) forms a cluster.

**
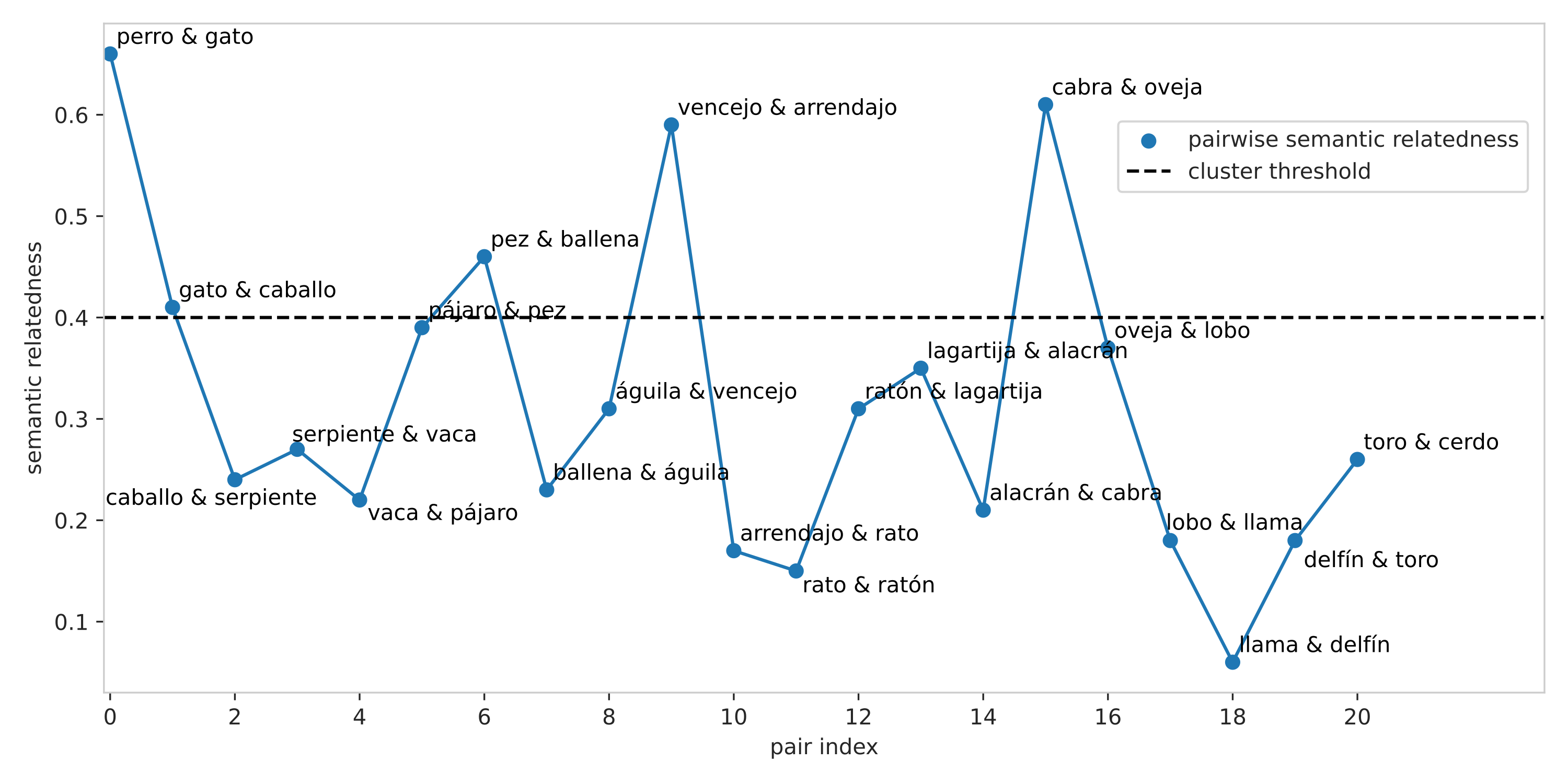
**

**Figure S6: Pairwise similarity of semantic VFT (Spanish).**

The figure depicts the pairwise semantic relatedness of all sequential word pairs from a semantic VFT in Spanish. Each series of words which shows a pairwise semantic relatedness above the fixed threshold (dashed line) forms a cluster.

**
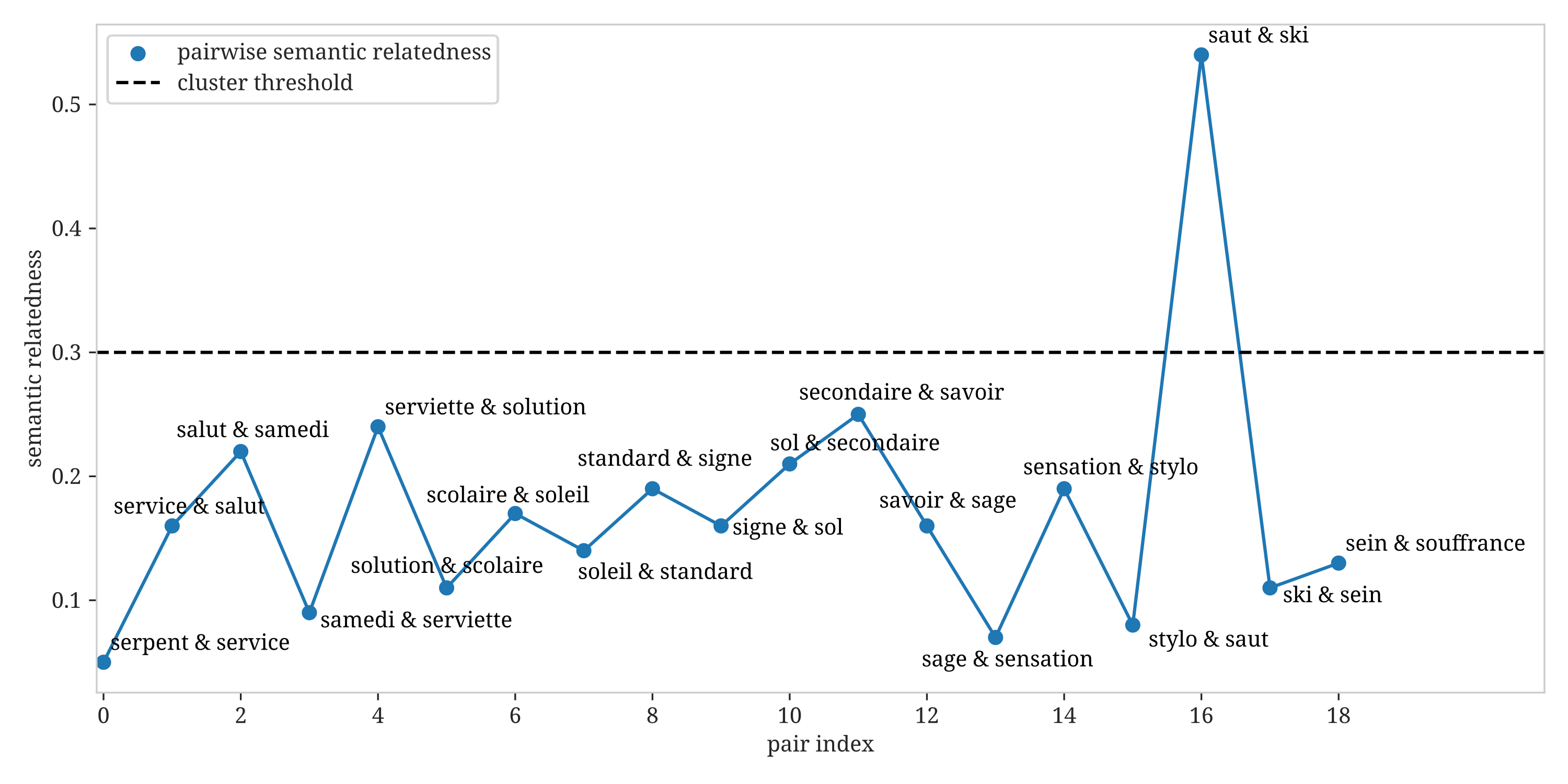
**

**Figure S7: Pairwise similarity of phonematic VFT (French).**

The figure depicts the pairwise semantic relatedness of all sequential word pairs from a phonematic VFT in French. Each series of words which shows a pairwise semantic relatedness above the fixed threshold (dashed line) forms a cluster.

**
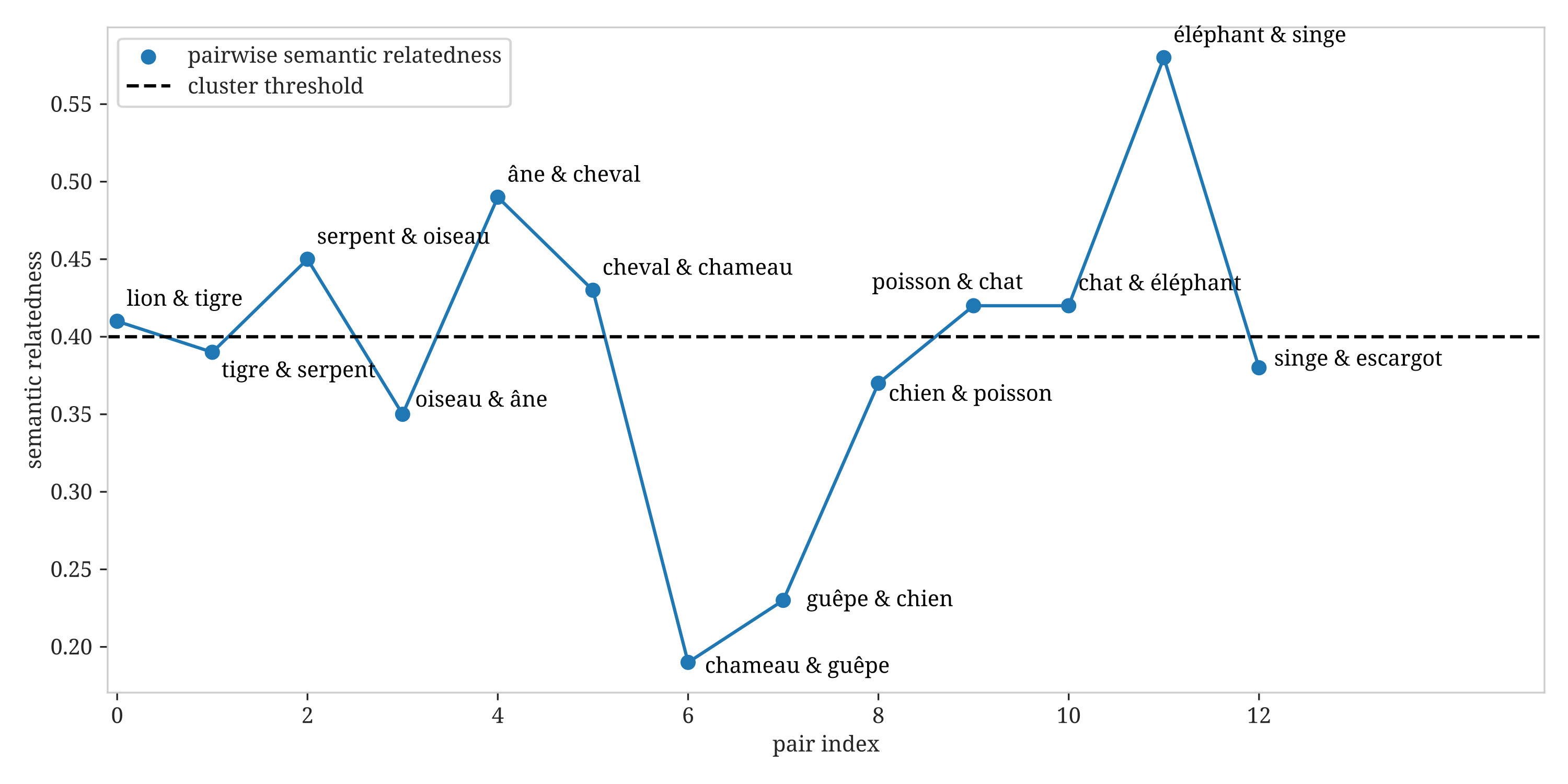
**

**Figure S8: Pairwise similarity of semantic VFT (French).**

The figure depicts the pairwise semantic relatedness of all sequential word pairs from a semantic VFT in French. Each series of words which shows a pairwise semantic relatedness above the fixed threshold (dashed line) forms a cluster.

| **Parameter** | **Rule-based clusters (female)** | **Rule-based clusters (male)** | **Rule-based**  **p-value** | **Semantic relatedness clusters (female)** | **Semantic relatedness clusters (male)** | **Semantic relatedness**  **p-value** |
| --- | --- | --- | --- | --- | --- | --- |
| **Total word count (mean, SD)** | 13.1  (± 4.7) | 11.7  (± 4.6) | 0.218 | 13.1  (± 4.7) | 11.7  (± 4.6) | 0.218 |
| **Mean cluster size (mean, SD)** | 0.3  (± 0.2) | 0.4  (± 0.5) | 0.149 | 0.2  (± 0.2) | 0.1  (± 0.1) | 0.108 |
| **Switches**  **(mean, SD)** | **10.9**  **(± 4.2)** | **8.7**  **(± 3.8)** | **0.030*** | 11.3  (± 4.3) | 10.4  (± 3.8) | 0.349 |
| **Mean sequential semantic relatedness**  **(mean, SD)** |  |  |  | 19.2%  (± 4.5%) | 18.7%  (± 4.3%) | 0.457 |

**Table S1: Phonematic VFT cluster characteristics compared by sex.**

Traditional (rule-based) and semantic relatedness cluster characteristics for the phonematic VFT separated for female and male patients. Additionally, p-values for comparisons are shown. Significant differences (p-values below 0.05) are indicated by bold font and a star (*). Abbreviations: SD: standard deviation

| **Parameter** | **List-based clusters (female)** | **List-based clusters (male)** | **List-based**  **p-value** | **Semantic relatedness clusters (female)** | **Semantic relatedness clusters (male)** | **Semantic relatedness p-value** |
| --- | --- | --- | --- | --- | --- | --- |
| **Total word count (mean, SD)** | 19.1  (± 5.4) | 19.8  (± 5.8) | 0.685 | 19.1  (± 5.4) | 19.8  (± 5.8) | 0.685 |
| **Mean cluster size (mean, SD)** | 0.9  (± 0.4) | 1.0  (± 0.6) | 1 | 0.9  (± 0.6) | 0.7  (± 0.6) | 0.111 |
| **Switches**  **(mean, SD)** | 10.3  (± 2.9) | 10.3  (± 3.7) | 0.767 | 10.8  (± 3.3) | 12.6  (± 4.7) | 0.261 |
| **Mean sequential semantic relatedness**  **(mean, SD)** |  |  |  | 37.8%  (± 4.5%) | 36.4%  (± 5.6%) | 0.376 |

**Table S2 Semantic VFT cluster characteristics compared by sex.**

Traditional (list-based) and semantic relatedness cluster characteristics for the semantic VFT separated for female and male patients. Additionally, p-values for comparisons are shown. Significant differences (p-values below 0.05) are indicated by bold font and a star (*). Abbreviations: SD: standard deviation

| **Parameter** | **Rule-based clusters (young)** | **Rule-based clusters (old)** | **Rule-based**  **p-value** | **Semantic relatedness clusters (young)** | **Semantic relatedness clusters (old)** | **Semantic relatedness p-value** |
| --- | --- | --- | --- | --- | --- | --- |
| **Total word count (mean, SD)** | 12.7  (± 4.7) | 11.9  (± 4.6) | 0.497 | 12.7  (± 4.7) | 11.9  (± 4.6) | 0.497 |
| **Mean cluster size (mean, SD)** | 0.3  (± 0.3) | 0.5  (± 0.5) | 0.159 | 0.2  (± 0.2) | 0.1  (± 0.1) | 0.089 |
| **Switches**  **(mean, SD)** | 9.9  (± 3.4) | 9.1  (± 4.7) | 0.245 | 10.5  (± 3.8) | 11.0  (± 4.3) | 0.610 |
| **Mean sequential semantic relatedness**  **(mean, SD)** |  |  |  | 19.7%  (± 4.4%) | 18.1%  (± 4.3%) | 0.114 |

**Table S3 Phonematic VFT cluster characteristics compared by age.**

Traditional (rule-based) and semantic relatedness cluster characteristics for the phonematic VFT separated for young and old patients. Patients were separated in two equal-sized groups by the median age in our patient cohort (63.5 years). Additionally, p-values for comparison between both age groups are shown. Significant differences (p-values below 0.05) are indicated by bold font and a star (*). Abbreviations: SD: standard deviation

| **Parameter** | **List-based clusters (young)** | **List-based clusters (old)** | **List-based**  **p-value** | **Semantic relatedness clusters (young)** | **Semantic relatedness clusters (old)** | **Semantic relatedness p-value** |
| --- | --- | --- | --- | --- | --- | --- |
| **Total word count (mean, SD)** | **21.4**  **(± 6.0)** | **17.5**  **(± 4.6)** | **0.013*** | **21.4**  **(± 6.0)** | **17.5**  **(± 4.6)** | **0.013*** |
| **Mean cluster size (mean, SD)** | 1.0  (± 0.6) | 0.9  (± 0.4) | 0.401 | 0.8  (± 0.6) | 0.7  (± 0.6) | 0.678 |
| **Switches**  **(mean, SD)** | 10.9  (± 3.9) | 9.6  (± 2.8) | 0.448 | 13.1  (± 5.1) | 10.9  (± 3.0) | 0.060 |
| **Mean sequential semantic relatedness**  **(mean, SD)** |  |  |  | 37.1%  (± 5.3%) | 36.5%  (± 5.4%) | 0.660 |

**Table S4 Semantic VFT cluster characteristics compared by age.**

Traditional (list-based) and semantic relatedness cluster characteristics for the semantic VFT separated for young and old patients. Patients were separated in two equal-sized groups by the median age in our patient cohort (63.5 years). Additionally, p-values for comparison between both age groups are shown. Significant differences (p-values below 0.05) are indicated by bold font and a star (*). Abbreviations: SD: standard deviation

| **Parameter** | **Rule-based clusters (early)** | **Rule-based clusters (late)** | **Rule-based**  **p-value** | **Semantic relatedness clusters (early)** | **Semantic relatedness clusters (late)** | **Semantic relatedness p-value** |
| --- | --- | --- | --- | --- | --- | --- |
| **Total word count (mean, SD)** | 11.2  (± 4.2) | 13.1  (± 4.8) | 0.097 | 11.2  (± 4.2) | 13.1  (± 4.8) | 0.097 |
| **Mean cluster size (mean, SD)** | 0.3  (± 0.5) | 0.4  (± 0.4) | 0.054 | 0.2  (± 0.1) | 0.2  (± 0.1) | 0.976 |
| **Switches**  **(mean, SD)** | 8.9  (± 3.7) | 10.0  (± 4.4) | 0.258 | **9.6**  **(± 3.4)** | **11.6**  **(± 4.3)** | **0.039*** |
| **Mean sequential semantic relatedness**  **(mean, SD)** |  |  |  | 19.1%  (± 4.3%) | 18.7%  (± 4.4%) | 0.741 |

**Table S5 Phonematic VFT cluster characteristics compared by disease duration.**

Traditional (rule-based) and semantic relatedness cluster characteristics for the phonematic VFT separated for early and late disease stage patients. Patients were separated in two equal-sized groups by the median disease duration in our patient cohort (6.0 years). Additionally, p-values for comparison between both disease duration groups are shown. Significant differences (p-values below 0.05) are indicated by bold font and a star (*). Abbreviations: SD: standard deviation

| **Parameter** | **List-based clusters (early)** | **List-based clusters (late)** | **List-based**  **p-value** | **Semantic relatedness clusters (early)** | **Semantic relatedness clusters (late)** | **Semantic relatedness p-value** |
| --- | --- | --- | --- | --- | --- | --- |
| **Total word count (mean, SD)** | 19.9  (± 5.8) | 19.3  (± 5.7) | 0.74 | 19.9  (± 5.8) | 19.3  (± 5.7) | 0.740 |
| **Mean cluster size (mean, SD)** | 0.9  (± 0.6) | 1.0  (± 0.5) | 0.584 | 0.8  (± 0.6) | 0.7  (± 0.6) | 0.572 |
| **Switches**  **(mean, SD)** | 11.0  (± 3.9) | 9.6  (± 3.0) | 0.156 | 12.3  (± 4.8) | 11.8  (± 4.0) | 0.992 |
| **Mean sequential semantic relatedness**  **(mean, SD)** |  |  |  | 36.6%  (± 5.8%) | 37.0%  (± 4.9%) | 0.578 |

**Table S6 Semantic VFT cluster characteristics compared by disease duration.**

Traditional (list-based) and semantic relatedness cluster characteristics for the semantic VFT separated for early and late disease stage patients. Patients were separated in two equal-sized groups by the median disease duration in our patient cohort (6.0 years). Additionally, p-values for comparison between both disease duration groups are shown. Significant differences (p-values below 0.05) are indicated by bold font and a star (*). Abbreviations: SD: standard deviation
